# Consumer-Based Wearable Activity Trackers Increase Physical Activity Participation: Systematic Review and Meta-Analysis

**DOI:** 10.2196/11819

**Published:** 2019-04-12

**Authors:** Katie-Jane Brickwood, Greig Watson, Jane O'Brien, Andrew D Williams

**Affiliations:** 1 School of Health Science College of Health and Medicine University of Tasmania Newnham Australia

**Keywords:** exercise, fitness trackers, telemedicine, meta-analysis

## Abstract

**Background:**

The range of benefits associated with regular physical activity participation is irrefutable. Despite the well-known benefits, physical inactivity remains one of the major contributing factors to ill-health throughout industrialized countries. Traditional lifestyle interventions such as group education or telephone counseling are effective at increasing physical activity participation; however, physical activity levels tend to decline over time. Consumer-based wearable activity trackers that allow users to objectively monitor activity levels are now widely available and may offer an alternative method for assisting individuals to remain physically active.

**Objective:**

This review aimed to determine the effects of interventions utilizing consumer-based wearable activity trackers on physical activity participation and sedentary behavior when compared with interventions that do not utilize activity tracker feedback.

**Methods:**

A systematic review was performed searching the following databases for studies that included the use of a consumer-based wearable activity tracker to improve physical activity participation: Cochrane Controlled Register of Trials, MEDLINE, PubMed, Scopus, Web of Science, Cumulative Index of Nursing and Allied Health Literature, SPORTDiscus, and Health Technology Assessments. Controlled trials of adults comparing the use of a consumer-based wearable activity tracker with other nonactivity tracker–based interventions were included. The main outcome measures were physical activity participation and sedentary behavior. All studies were assessed for risk of bias, and the Grades of Recommendation, Assessment, Development, and Evaluation system was used to rank the quality of evidence. The guidelines of the Preferred Reporting Items for Systematic Reviews and Meta-Analyses statement were followed. A random-effects meta-analysis was completed on the included outcome measures to estimate the treatment effect of interventions that included an activity tracker compared with a control group.

**Results:**

There was a significant increase in daily step count (standardized mean difference [SMD] 0.24; 95% CI 0.16 to 0.33; *P*<.001), moderate and vigorous physical activity (SMD 0.27; 95% CI 0.15 to 0.39; *P*<.001), and energy expenditure (SMD 0.28; 95% CI 0.03 to 0.54; *P*=.03) and a nonsignificant decrease in sedentary behavior (SMD −0.20; 95% CI −0.43 to 0.03; *P*=.08) following the intervention versus control comparator across all studies in the meta-analyses. In general, included studies were at low risk of bias, except for performance bias. Heterogeneity varied across the included meta-analyses ranging from low (I^2^=3%) for daily step count through to high (I^2^=67%) for sedentary behavior.

**Conclusions:**

Utilizing a consumer-based wearable activity tracker as either the primary component of an intervention or as part of a broader physical activity intervention has the potential to increase physical activity participation. As the effects of physical activity interventions are often short term, the inclusion of a consumer-based wearable activity tracker may provide an effective tool to assist health professionals to provide ongoing monitoring and support.

## Introduction

### Physical Activity and Sedentary Behavior

There is significant evidence to support the varied physical and mental health benefits of participation in regular physical activity [1-4]. In addition, regular participation in physical activity plays an important role in maintaining functional independence into aging and decreases the risk of morbidity and mortality [5]. Despite the well-known benefits of physical activity participation, 31% of adults worldwide are insufficiently active as they do not meet the minimum recommendations of at least 30 min of moderate-intensity physical activity on at least 5 days every week, 20 min of vigorous-intensity physical activity on at least 3 days every week, or an equivalent combination achieving 600 metabolic equivalent min per week [6]. In addition, sedentary behavior, which is defined as any waking behavior while in a sitting, reclining, or lying position [7], is independently associated with poor health outcomes, including all-cause and cardiovascular disease mortality [8,9].

### Traditional Interventions to Increase Physical Activity Participation

The use of structured lifestyle interventions is reported to be effective in increasing physical activity participation and reducing the progression of chronic diseases [10-12]. In addition, lifestyle interventions have shown to be effective in reducing sedentary behavior [13]. Traditionally, structured lifestyle interventions utilize group or individual education, behavior change techniques, self-monitoring, the provision of written information materials, and/or telephone counseling. Interventions utilizing these methods have shown to be effective at increasing physical activity participation in the short term [12,14]; however, evidence regarding their long-term effectiveness is limited [15-17]. In addition, these types of interventions are often labor and resource intensive [18].

### Wearable Activity Trackers

Consumer-based wearable activity trackers are now readily available and can provide individuals with the ability to objectively monitor their physical activity levels. In addition, when combined with the use of smartphone and computer apps, they may assist users through a range of motivational and tracking tools to better manage their personal health [19]. In addition to providing real-time feedback relating to daily steps and energy expenditure, consumer-based wearable activity trackers have the potential to provide specific, tailored feedback through specifically designed algorithms or by health professionals. This type of emerging technology may provide an alternative means of providing ongoing support and motivation to individuals both looking to increase their activity levels or to maintain activity levels following a structured lifestyle intervention [20]. Moreover, consumer-based wearable activity trackers may assist in reducing the resource and time burden associated with traditional methods of providing ongoing support. Randomized controlled trials have shown that these devices have promise in relation to increasing physical activity participation [21,22]; however, participant numbers in individual studies tend to be low, making it difficult to adequately assess the benefits of these devices. Furthermore, there is limited research relating to their long-term adherence and effectiveness. This systematic review and meta-analysis aimed to review the effects of interventions that utilize consumer-based wearable activity trackers compared with a nonactivity tracker–based control group on physical activity participation in adults.

## Methods

### Search Method

The following Web-based databases were searched using a combination of Medical Subject Headings (MeSH) and free text terms: CENTRAL, MEDLINE, PubMed, Scopus, Web of Science, CINHAL, SPORTDiscus, and Health Technology Assessment. Search strategies were developed relating to the 2 primary concepts of the review: the use of a consumer-based wearable activity tracker and altering physical activity participation. To identify studies that included the use of a consumer-based wearable activity tracker, we used search terms including *Activity Tracker*, *Wearable device*, and *Fitness Tracker* (MeSH). Search terms used to identify studies that focused on altering physical activity participation included *Physical Fitness* (MeSH), *Sedentary Lifestyle* (MeSH), *Step Count*, and *Behaviour Change*. Each database was searched from inception to March 15, 2017, with no language restrictions. Search strategies were adapted for each database as necessary. A full search strategy is available in Multimedia Appendix 1. Reference lists of retrieved articles were checked, and citation searches were performed on key articles. Authors were contacted for additional information where necessary. The search was limited to human studies.

### Inclusion and Exclusion Criteria

Published and unpublished controlled trials of adults (aged over 18 years) that utilized a consumer-based wearable activity tracker were included in this review. The effect of consumer-based wearable activity trackers on physical activity participation and sedentary behavior was assessed. For the purpose of this review, consumer-based wearable activity trackers were defined as an electronic device that monitors physical activity and provides automated real-time feedback and may also include interactive behavior change tools via a smartphone or Web-based platform. Consumer-based tracker refers to an activity tracker that is available for purchase to the general public and therefore excludes laboratory-based or research-specific devices. Wearable tracker refers to a device that is easily worn and removed and does not require specialized equipment such as a harness or adhesive dressings.

Studies that included the use of a consumer-based wearable activity tracker as either the basis of the intervention or as a component of a multifaceted intervention were included. Studies that included the use of established behavioral change techniques such as group or individual counseling or information sessions, financial incentives, or telephone counseling were classified as multifaceted interventions. Interventions that included tools such as regular emails, text messages, online algorithms, or smartphone apps were classified as *wearable-based* interventions.

Studies that compared consumer-based wearable activity trackers with exercise-based interventions (eg, exercise groups), nonexercise interventions (eg, group education programs), and routine (usual) care were included. Control groups that also utilized a consumer-based wearable activity tracker were included; provided feedback from the activity tracker was blinded to the participant. Studies that utilized consumer-based wearable activity trackers for the entirety of the intervention or as a follow-up component to a structured lifestyle intervention were included as were studies examining the effect of consumer-based wearable activity trackers on sedentary behavior.

Review articles, validity, reliability studies, and conference abstracts were excluded. Acceptability and feasibility studies were included provided data relating to physical activity participation was included. The authors of identified ongoing studies were contacted to obtain study progress and request available results for inclusion in the meta-analysis.

### Data Extraction

Titles and abstracts were screened in 4 steps: removal of duplicates, by title, by abstract, and by full text. Article titles and abstracts were systematically screened based on the predetermined exclusion criteria (Multimedia Appendix 2). Potentially eligible papers were retrieved by the primary author (KB). All manuscripts identified as requiring full-text review were reviewed independently by 2 authors (KB and AW) according to the exclusion criteria. A third reviewer (GW) resolved any conflicts. The data extraction tool in Covidence (Veritas Health Innovation Ltd, VIC 3000, Australia) online software [23] was used with data extraction performed by both authors (KB and AW) individually and differences resolved by consensus.

### Risk of Bias and Quality Assessment

Two reviewers (KB and AW) assessed each study independently for risk of bias using Covidence online software [23] across 7 domains [24]. Each domain was scored as low, unclear, or high risk of bias. Disagreement was resolved by consensus. The following domains were assessed:

- Sequence generation: Was the method used to generate the allocation sequence appropriate to produce comparable groups? The risk of bias was rated as unclear if methods were not accurately described.
- Allocation sequence generation: Was the method used to conceal the allocation sequence appropriate to prevent allocation being known? The risk of bias was rated as unclear if methods were not accurately described.
- Blinding of participants and personnel: Were participants and study personnel blinded to the group allocation? Although this domain was included in the risk of bias assessment, it is important to note that because of the type of studies included, blinding of participants is not feasible, and therefore, all studies were assessed as high risk of bias for this domain.
- Blinding of outcome assessment: Was the outcome measure objective or subjective? If a subjective measure was used, the risk of bias was assessed as high. If an objective measure was used, the risk of bias was assessed as low as objective measures are less likely to be influenced by a lack of blinding.
- Incomplete outcome data: Were incomplete outcome data adequately addressed? Was the analysis an intention-to-treat analysis or were missing data imputed appropriately?
- Selective outcome reporting: Were outcomes prespecified in a study protocol or trial registration and reported as specified?
- Other sources of bias: Were there other sources of bias not previously mentioned, such as author conflicts of interest?

The Grades of Recommendation, Assessment, Development, and Evaluation (GRADE) system was used to rank the quality of evidence for each study using GRADEprofiler Guideline Development Tool online software [25] in conjunction with Chapter 12.2 of the *Cochrane Handbook for Systematic Reviews of Interventions* [24]. The GRADE approach uses 5 considerations (study limitations, consistency of effect, imprecision, indirectness, and publication bias) to assess the quality of evidence for each outcome. The following criteria are used for assigning a grade of evidence:

- High: further research is very unlikely to change our confidence in the estimate of effect.
- Moderate: we are moderately confident the true effect is likely to be close to the estimate of effect, but there is a possibility that it is substantially different.
- Low: our confidence in the effect estimate is limited; the true effect may be substantially different from the estimate of the effect.
- Very low: we have very little confidence in the effect estimate; the true effect is likely to be substantially different from the estimate of the effect.

The grade of evidence was downgraded once if:

- More than 25% (n=7) of included studies were at high risk of bias in any criteria (study limitations)
- Heterogeneity was statistically significant and the I^2^ value was more than 40% (inconsistency)
- There were differences between included studies in methodological factors such as intervention types and length, included age ranges, and included study populations (indirectness)
- Observed confidence intervals were wide because of small sample sizes (imprecision)
- There was direct evidence of publication bias.

### Data Synthesis

Due to the variability of the included studies, random-effects meta-analyses [26] were performed on the following physical activity outcomes using Review Manager (RevMan) [27]: daily step count, minutes per week spent in moderate and vigorous physical activity (MVPA; min/week), energy expenditure (kcal/week), and minutes per day spent in sedentary behavior (min/day). Data presented as minutes of MVPA per day were multiplied by 7 to calculate minutes of MVPA per week to allow for greater transferability to current physical activity guidelines. Where sedentary behavior data were presented as hours per day, values were divided by 60 to obtain minutes per day. Each meta-analysis compared the results of the intervention group(s) with the study-specific control group. Studies that included multiple intervention groups were entered multiple times, with each intervention group compared against the control group. Studies that included interventions that did not utilize a consumer-based wearable activity tracker were not included in the meta-analysis. Data presented as least-squares mean, SE, or 95% CI were converted to SD using the RevMan calculator. Due to the range of data presentation formats of included studies, all meta-analyses were presented as standardized mean difference (SMD) to accommodate for adjusted and unadjusted means [24]. Mean and SD were requested from authors where data were presented as median and interquartile ranges (IQR) based on the suggestion that estimated mean and SD can be used in a meta-analysis [28]. Authors of studies that presented data in a graphical format were contacted to obtain exact values. Heterogeneity was assessed using I^2^ for each meta-analysis.

Where intervention effects were reported using SMD, the reported values were converted into a meaningful value using the pooled SD of studies that reported end point values. Studies that presented mean change data or in which SD was estimated based on IQR were not included in the pooled SD calculation.

## Results

### Study Selection

**Figure 1:**
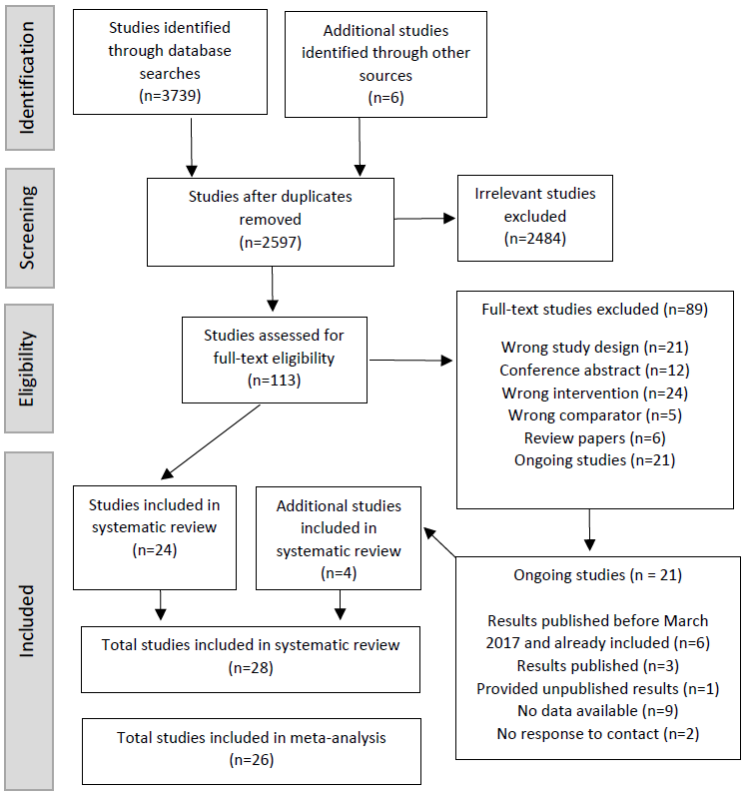
Flowchart of study selection. Studies with results published and authors that provided unpublished results were included in the systematic review.

The database search was completed during March 2017, with article collection and screening conducted in April to June 2017. A total of 3739 studies were retrieved from the search strategy, with a further 6 studies identified through reference checks. No non-English papers were identified. A total of 1148 duplicates were removed; 2597 studies were screened by title and abstract, with 2484 studies removed as they did not meet the inclusion criteria. The remaining 113 studies were assessed for full-text eligibility, with 89 studies excluded. A total of 28 randomized controlled trials were included in this systematic review [21,22,29-54]. Authors of study protocols were contacted to obtain study progress and results if available. As of December 2017, 3 authors had since published study results [22,36,39] and 1 author [33] had provided unpublished results, which were therefore included in the current systematic review and meta-analysis. Two studies [49,54] were excluded from the meta-analysis. One study [54] was excluded because of all the data being presented in a graphical format. The other study [49] was not included in the meta-analysis as physical activity data were reported in *activity units*. One additional study [38] was excluded from the MVPA meta-analysis because of the graphical representation of data but was not excluded from all meta-analyses as other data were presented in a tabular format. Figure 1 outlines the screening process, including the status of ongoing studies.

### Study Characteristics

A total of 3646 participants across 9 countries were included, with a mean age ranging from 17.9 years to 79.5 years. Included studies were all published between 2007 and 2018. One study was published only as a protocol paper with unpublished results provided by the author [33]. Thirteen studies included young adults (≤39.9 years) [30-34,37,38,42,44,46-48,50], 15 included middle-aged adults (40-64.9 years) [21,22,29,35,36,39-41, 43,45,51-54], and 1 study included adults aged over 65 years [49]. Furthermore, 17 studies specified that the participant must have regular access to the internet, a computer, tablet, and/or smartphone [21,30,33,35-37,39,41-47,51-53], with 3 studies requiring participants to be proficient at using the internet and/or smartphones [21,22,45].

Comparator interventions differed across the studies and ranged from maintenance of usual lifestyle [31,44,53], waitlist [30,36,39,40,52], wearing of a consumer-based wearable activity tracker but blinded to feedback [22,38,41,49,50], use of a standard pedometer [21], standard behavioral group-based interventions [29,37,43,45,46,51], telephone counseling [35,37], use of a smartphone app [33,42], and the provision of education materials through mixed media (emails, text message, and written) [21,32,34,47,48,54].

The way in which a consumer-based wearable activity tracker was incorporated into the interventions of included studies ranged from forming the basis of the intervention (wearable-based) [22,31,33,34,38,41,42,44,47,48,54] to being used as a monitoring tool as part of a broader intervention (multifaceted) [21,29,30,32,34-37,39,40,43,45-47,49-54]. Overall, 8 studies included more than 1 intervention group [34,43,45-47,52-54]. In addition, 4 studies included either an unstructured follow-up phase [34,42] or additional intervention phase utilizing a nonblinded activity tracker for all participants [41,50]. Data from these phases were not included in this review as they did not meet the eligibility criteria. Moreover, 17 studies reported activity tracker adherence data [21,22,30,32,34,35, 37,38,40,41,43-45,50-52,54], with 13 of these studies [21,22,30,34,35,40,41,43,44,50-52,54] reporting activity tracker wear on at least 50% of the study intervention days. Furthermore, 4 studies [32,37,38,45] reported low activity tracker wear time, with 1 study [32] reporting that all participants had ceased wearing their activity tracker by the end of the intervention. All studies included some form of behavioral change techniques ranging from basic techniques such as the provision of feedback and goal setting to interventions based on the Coventry, Aberdeen and London-Refined taxonomy [55], social cognitive theory [56], and social determination theory [57].

The initial search resulted in 21 protocol publications being identified [33,58-77]. Of the identified protocol publications, 6 had published results available, which were also identified as part of the initial search strategy [59,71,72,74,75,77]. Results from 3 protocol publications [61,65,68] were published after the initial search and were subsequently included [22,36,39]. One author provided unpublished results [33], 2 protocol authors did not return contact [66,76], and 9 authors were still collecting data or preparing manuscripts [58,60,62-64,67,69,70,73].

### Accuracy and Reliability of Included Consumer-Based Wearable Activity Trackers

Included studies utilized a range of consumer-based wearable activity trackers, including various Fitbit models [21,29,33-36,49,50] (Fitbit, San Francisco, CA), Jawbone UP 24 [22,30,40,42] (Jawbone, San Francisco, CA), Gruve [31] (Gruve Solution MUVE, Inc, USA), LumoBack [32] (Lumo BodyTech, Inc, Palo Alto, California, USA), various BodyMedia models [37,43,45,46,51,54] (BodyMedia, Pittsburgh PA), Polar Active [38] (Polar Electro, Finland), Fitbug [41] (Chicago IL), Pebble+ [44] (Fitlinxx Inc), Fitmeter [47] (FitLife, Suwon, Korea), Personal Activity Monitor [48] (PAM BV, Doorwerth, the Netherlands), and Withings Pulse [52] (Cambridge, MA). One study did not specify the brand of consumer-based wearable activity tracker utilized [53]. Fitbit One, Zip and Charge HR, Jawbone Up, LumoBack, and Withings Pulse have all demonstrated excellent test-retest reliability for step count (Intraclass Correlation Coefficient [ICC] >.90) [78-80]; however, a recent review into the use of Fitbit activity trackers suggests that steps are overestimated in free-living conditions [81]. The Polar Active has shown to correlate (*r*^2^=0.74) with the doubly labeled water technique for assessing energy expenditure during military training, which related to the study setting [82]. The Bodymedia Sensewear showed good reliability during outdoor walking (ICC=0.82); however, poor reliability was observed during various treadmill walking speeds (ICC=0.18 to 0.27) [80]. An earlier version of the Pebble+, the ActiPed by FitLinxx has demonstrated a high level of accuracy for step count (−1.30%) and good reliability (ICC=0.85) [83]; however, currently, there are no validity or reliability data for the Pebble+. The PAM has shown similar validity to the ActiGraph accelerometer (*r*^2^=0.95) and good reliability (ICC=0.80) [84]. There is currently limited reliability and validity data relating to the Fitmeter; however, it has been reported that the Fitmeter does correlate with gas analyzer measures for energy expenditure (*r*^2^=0.82) [85]. The Gruve monitor has been shown to be accurate in measuring sedentary and walking activities when compared with a gold standard system (*r*^2^=0.98) [86] and is recommended for use in interventions aiming to reduce sedentary behavior [87]. No validation or reliability data could be found relating to the Fitbug Orb.

### Study Outcomes

Physical activity behavior measures included number of steps taken per day [21,22,29,30,32,34,40-42,44,50,54], minutes spent in MVPA per week [21,29,30,33-37,39,48,51,53], and energy expenditure [43,45-47,52]. Sedentary behavior was reported by 8 studies with data presented as hours per day [31,37,38], minutes per day [33,39,40], minutes per 16 hours [32], or percentage of total day spent sitting [29,37]. A summary of outcome measures for all included studies is included in Table 1. A detailed summary of included studies is available in Multimedia Appendix 3.

### Risk of Bias

**Figure 2:**
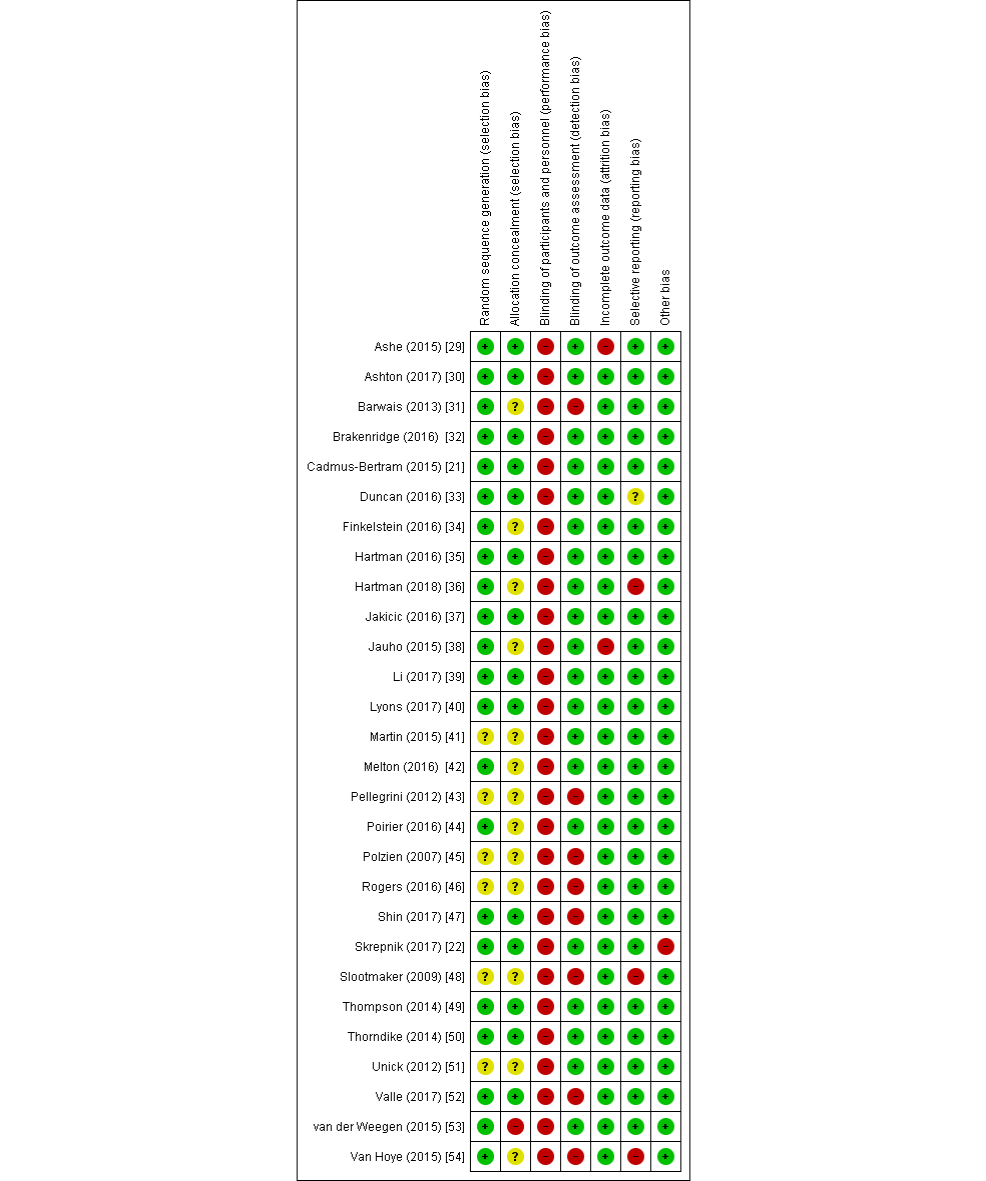
Risk of bias summary: review authors’ judgments about each risk of bias item for each included study. Green symbols represent a low risk of bias, yellow symbols represent an unclear risk of bias, and red symbols represent a high risk of bias.

Risk of bias judgments for each included study are presented in Figure 2. One study [53] was assessed as high risk of selection bias because of the randomization of practices rather than individuals. All studies were assessed as high risk of bias for performance bias because of the nature of the intervention and control conditions making blinding impossible. Blinding of outcome assessors (detection bias) was assessed as high risk for 7 studies [31,43,45-48,52] because of the use of subjective outcome measures. One further study [54] was also assessed as a high risk of detection bias as participants were provided with activity level feedback at each assessment with comparisons with previous results. The management of incomplete outcome data was assessed as high risk in 2 studies [29,38]. In addition, 3 studies [36,48,54] were assessed as high risk for selective outcome reporting, and 1 study [22] was judged as high risk for other sources of bias because of conflicts of interest declared by the authors. Publication bias was assessed for daily step count and MVPA with no bias identified. Publication bias was unable to be assessed for other outcome measures because of less than 10 included studies.

### Meta-Analysis Results

A total of 26 studies were included across all meta-analyses [21,22,29-48,50-53]. Results were primarily presented as mean and SD or 95% CI, or as mean change and SD, SE, or 95% CI. Two studies [48,52] presented data as median and IQRs, suggesting that the data were not normally distributed. Authors of both the studies were contacted, and they provided mean and SD data, and the studies were subsequently included in the meta-analyses [28]. One study [54] was excluded from the meta-analyses as all data were presented in a graphical format. The authors were contacted for results, but no return contact was received. An additional study was excluded [49] because of data being presented as *activity units*.

### Physical Activity Participation

#### Steps

**Figure 3:**
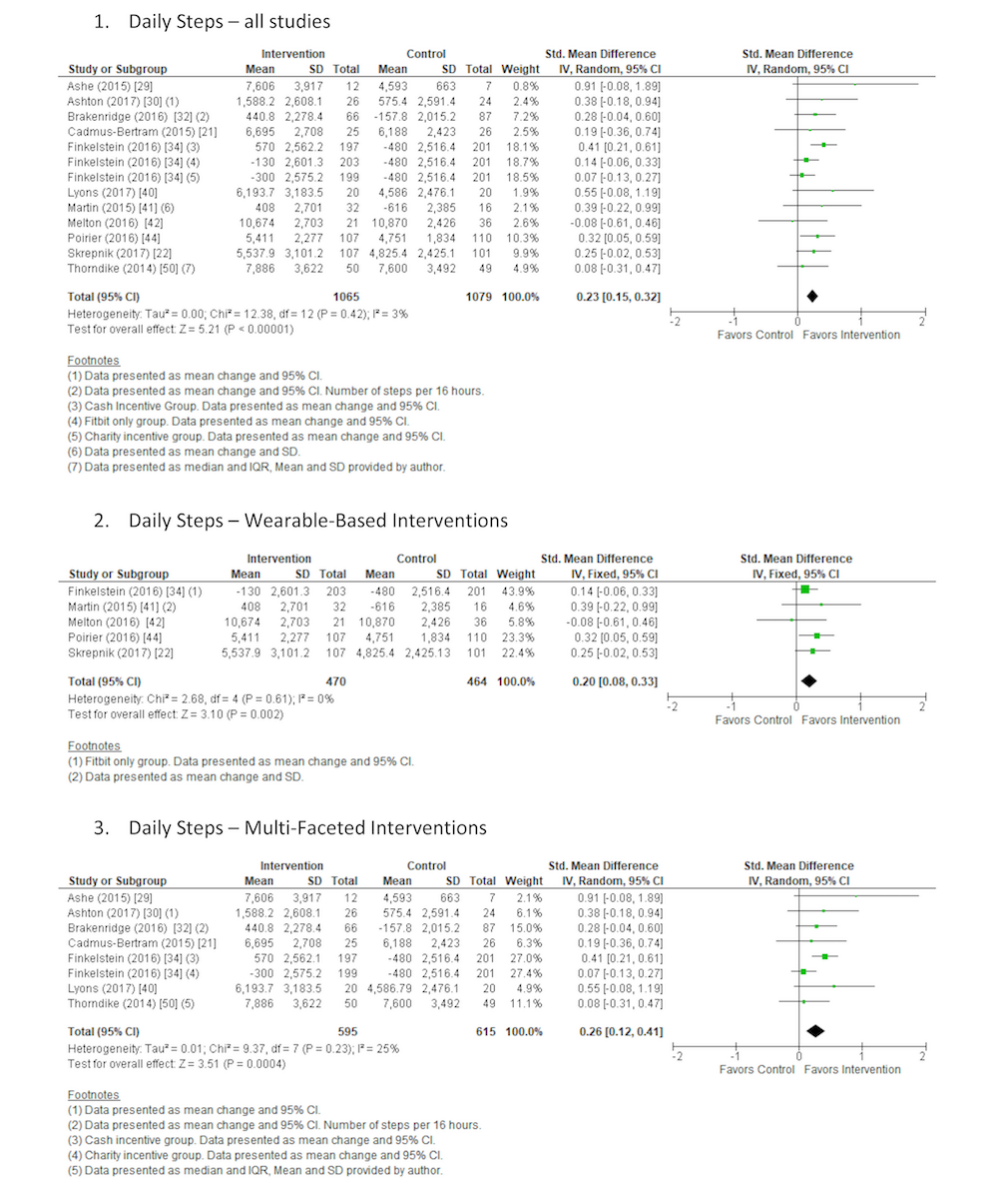
Forest plot of standardized mean difference of steps per day in studies comparing an intervention that included a consumer-based wearable activity tracker with a control group that did not utilize a consumer-based wearable activity tracker. Subgroup analysis was completed on studies that included wearable-based interventions compared with control and multifaceted interventions compared with control. Green square indicates the standardized mean difference for each individual study. Black square indicates the overall standardized mean difference for all studies.

Overall, 12 studies reported changes in the number of steps taken by participants [21,22,29,30,32,34,40-42,44,50,54]. A random-effects meta-analysis using SMD was performed on 11 studies as 1 study [54] was excluded because of graphical presentation of data. Step data were objectively measured using a range of accelerometers or pedometers. There was a significant increase in step count following the intervention versus control comparator (SMD 0.23; 95% CI 0.15 to 0.32; *P*<.001; Figure 3) across all studies in the meta-analysis, representing an approximate increase of 627 steps (95% CI 417 to 862 steps) per day. Heterogeneity was low [88] and nonsignificant (I^2^=3%; *P*=.42). We judged the quality of evidence for consumer-based wearable activity trackers to increase the daily number of steps as low. The quality of the evidence was rated as being low based on being downgraded twice, once because of the high risk of bias identified in the included studies and once because of the level of indirectness associated with the broad range of included interventions, comparators, populations, and settings. The summary of findings table for all outcome measures is available in Multimedia Appendix 4.

Further subgroup analysis was completed, separating the included studies into interventions that were wearable-based [22,34,41,42,44] and those that were multifaceted [21,29,30,32,34,40,50]. A significant increase in daily step count following the intervention versus control comparator was observed in both wearable-based (SMD 0.20; 95% CI 0.08 to 0.33; *P*=.002; Figure 3) and multifaceted (SMD 0.26; 95% CI 0.12 to 0.41; *P*>.001; Figure 3) meta-analyses. This is representative of an approximate increase of 475 steps (95% CI 190 to 784 steps) per day and 685 steps (95% CI 316 to 1080 steps) per day, respectively. Low and nonsignificant heterogeneity was observed in both subgroup analyses (I^2^=0%; *P*=.61 and I^2^=25%; *P*=.23, respectively).

**Table 1:** Outcome measures of physical activity participation and sedentary behavior.

Study	Outcome measures	Outcome measurement instrument	Objective/subjective
Ashe et al, 2015 [29]	Steps/day, MVPA^a^ (min/day), and sitting time (%)	ActiGraph GT3X accelerometer	Objective
Ashton et al, 2017 [30]	Steps/day and MVPA (min/week)	Yamax Digiwalker SW200 and Godin Leisure-Time Exercise Questionnaire	Objective and subjective
Barwais et al, 2013 [31]	Sitting time (hours/day)	7-day Sedentary and Light Intensity Physical Activity Log	Subjective
Brakeridge et al, 2016 [32]	Steps/day and sitting time (min/16 hours)	ActivPal activity monitor	Objective
Cadmus-Bertram et al, 2015 [21]	Steps/day and MVPA (min/week)	ActiGraph GT3X accelerometer	Objective
Duncan et al, 2016 [33]	MVPA (min/week) and sitting time (min/day)	Geneactiv accelerometer	Objective
Finkelstein et al, 2016 [34]	Steps/day and MVPA (min/week)	ActiGraph GT3X accelerometer	Objective
Hartman et al, 2016 [35]	MVPA (min/day)	ActiGraph GT3X accelerometer	Objective
Hartman et al, 2018 [36]	MVPA (min/day)	ActiGraph GT3X accelerometer	Objective
Jakicic et al, 2016 [37]	MVPA (min/week) and sitting time (hours/day)	Sensewear Pro Armband	Objective
Jauho et al, 2015 [38]	MVPA (min/day)^a^ and sitting time (hours/day)	Polar Active (as used in intervention)	Objective
Li et al, 2017 [39]	MVPA ≥3 metabolic equivalents (min/day) and sitting time (min/day)	Sensewear Mini Armband	Objective
Lyons et al, 2017 [40]	Steps/day and sitting time (min/day)	ActivPal activity monitor	Objective
Martin et al, 2015 [41]	Steps/day	Fitbug Orb accelerometer (as used in intervention)	Objective
Melton et al, 2016 [42]	Steps/day	ActiGraph GT3X accelerometer	Objective
Pellegrini et al, 2012 [43]	Energy expenditure (kcal/week)	PPAQ^b^	Subjective
Poirier et al, 2016 [44]	Steps/day	Pebble+ (as used intervention)	Objective
Polzien et al, 2007 [45]	Energy expenditure (kcal/week)	PPAQ	Subjective
Rogers et al, 2016 [46]	Energy expenditure (kcal/week)	PPAQ	Subjective
Shin et al, 2017 [47]	Energy expenditure (kcal/week)	International Physical Activity Questionnaire Short-Form	Subjective
Skrepnik et al, 2017 [22]	Steps/day	Jawbone UP 24 (as used in intervention)	Objective
Slootmaker et al, 2009 [48]	MVPA (min/week)	The Activity Questionnaire for Adolescents and Adults	Subjective
Thompson et al^c^, 2014 [49]	Activity units	Research-grade triaxial accelerometer	Objective
Thorndike et al, 2014 [50]	Steps/day	Fitbit (as used in intervention)	Objective
Unick et al, 2012 [51]	MVPA (min/week)	Sensewear Armband	Objective
Valle et al, 2017 [52]	Energy expenditure (kcal/week)	PPAQ	Subjective
van der Weegen et al, 2015 [53]	MVPA (min/day)	Personal Activity Monitor accelerometer	Objective
Van Hoye^c^ et al, 2015 [54]	Steps/day	Sensewear Armband (as used in intervention)	Objective

^a^MVPA: moderate and vigorous physical activity.

^b^PPAQ: Paffenbarger Physical Activity Questionnaire.

^c^Study not included in meta-analysis.

#### Moderate and Vigorous Physical Activity

**Figure 4:**
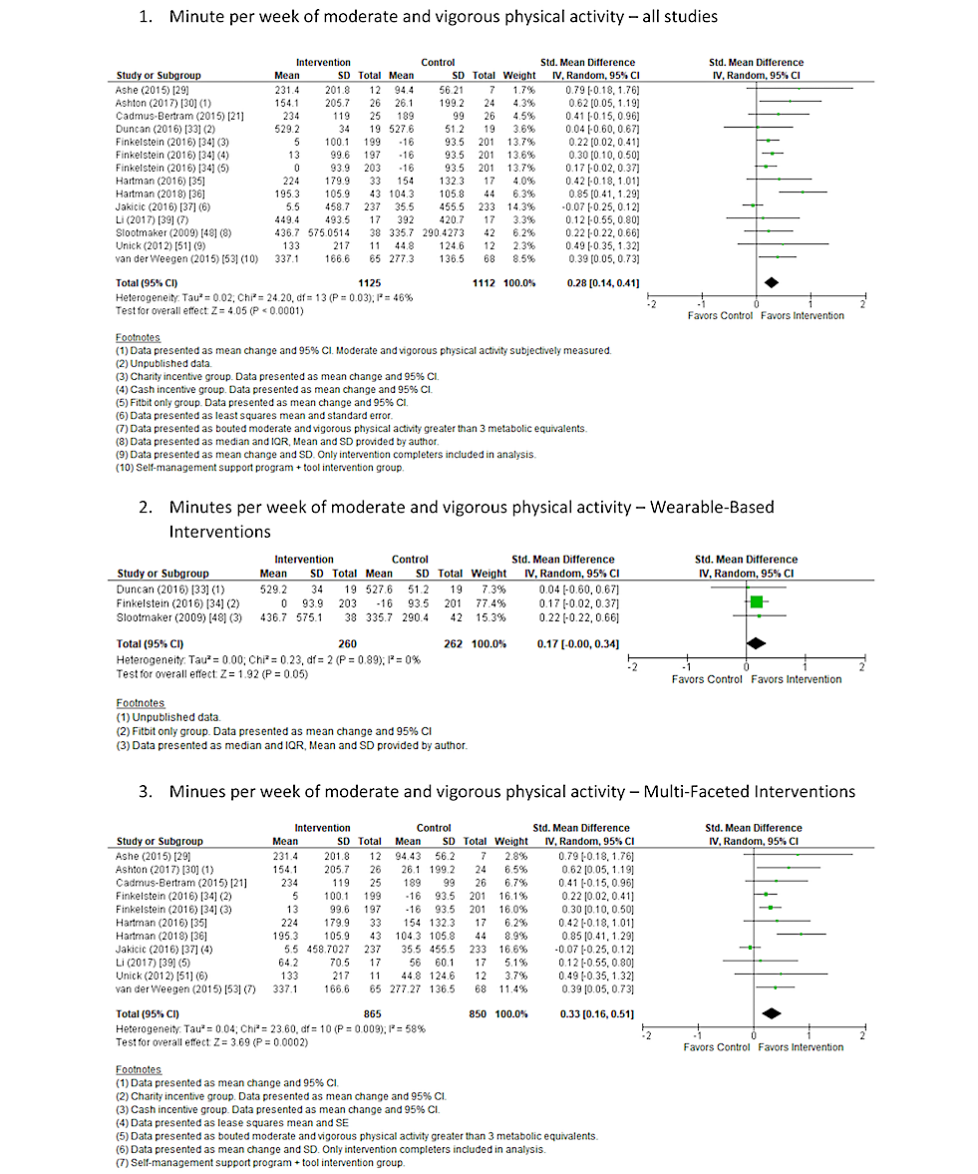
Forest plot of standardized mean difference of time spent in moderate and vigorous physical activity per week in studies comparing an intervention that included a consumer-based wearable activity tracker with a control group that did not utilize a consumer-based wearable activity tracker. Subgroup analysis was completed on studies that included wearable-based interventions compared with control and multifaceted interventions compared with control. Green square indicates the standardized mean difference for each individual study. Black square indicates the overall standardized mean difference for all studies.

A total of 15 studies measured levels of MVPA [21,29-31,33-39,48,51,53,54]. Moreover, 2 studies [38,54] presented data in a graphical format and 1 study [31] presented results for MVPA, separately. A random-effects meta-analysis using SMD was performed on the 12 remaining studies [21,29,30,33-37,39,48,51,53]. Of the included studies, 10 measured MVPA objectively through a range of accelerometers, and 2 studies [30,48] used subjective self-reported measures of MVPA. There was a significant increase in minutes per day spent in MVPA following the intervention versus control comparator (SMD 0.28; 95% CI 0.14 to 0.41; *P*<.001; Figure 4) across all studies in the meta-analysis with moderate and significant (I^2^=46%, *P*=.03) heterogeneity observed. These findings represent an approximate increase of 75 min (95% CI 42 to 109 min) per day of MVPA. The quality of the evidence was rated as very low based on being downgraded 3 times, once because of the high risk of bias identified, once because of the level of inconsistency associated with the observed heterogeneity, and once because of the level of indirectness associated with the broad range of included interventions, comparators, populations, and settings.

Further subgroup analysis was completed on studies that included wearable-based interventions [33,34,48] and multifaceted interventions [21,29,30,34-37,39,51,53]. There was a nonsignificant increase in minutes per day spent in MVPA following the intervention versus control comparator (SMD 0.17; 95% CI −0.00 to 0.34; *P*=.05; Figure 4) across studies included in the wearable-based meta-analysis, representing an approximate increase of 40 min (95% CI 0 to 80 min) per day of MVPA. Nonsignificant and low heterogeneity was observed (I^2^=0%; *P*=.89). A significant increase was observed in minutes per day spent in MVPA following the intervention versus control comparator (SMD 0.33; 95% CI 0.16 to 0.51; *P*<.001; Figure 4) across studies included in the multifaceted meta-analysis. This represents an approximate increase of 92 min (95% CI 45 to 142 min) per day of MVPA. Observed heterogeneity for studies included in the multifaceted intervention was high and significant (I^2^=58%; *P*=.009).

#### Energy Expenditure

**Figure 5:**
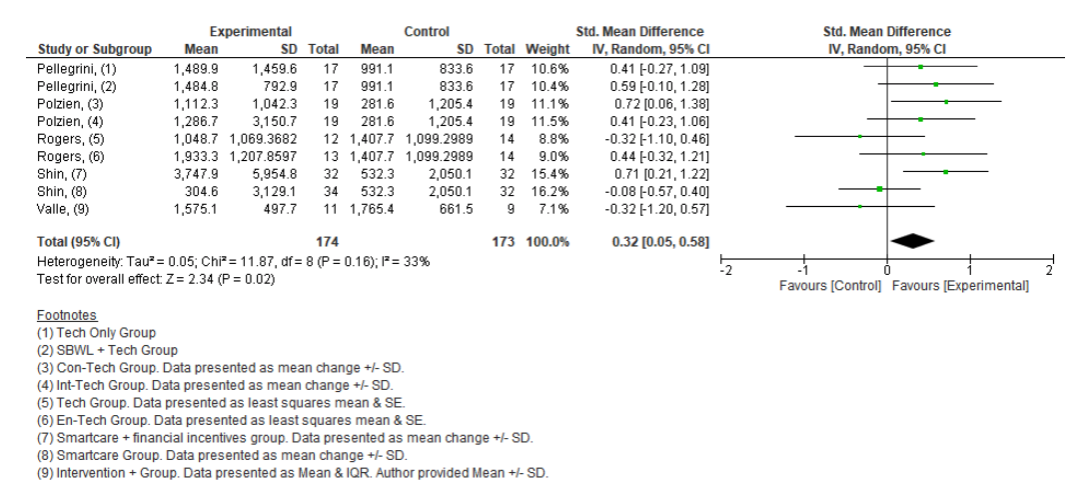
Forest plot of standardized mean difference of energy expenditure in studies comparing an intervention that included a consumer-based wearable activity tracker with a control group that did not utilize a consumer-based wearable activity tracker. Green square indicates the standardized mean difference for each individual study. Black square indicates the overall standardized mean difference for all studies.

Overall, 5 studies [43,45-47,52] reported physical activity levels in terms of energy expenditure, expressed as kcal per week. The Paffenbarger Physical Activity Questionnaire or the International Physical Activity Questionnaire were utilized in the included studies to obtain self-reported physical activity levels. A random effects meta-analysis using SMD performed on the five included studies showed a significant increase in energy expenditure following the intervention versus control comparator (SMD 0.32; 95%CI 0.05 to 0.58; *P*=.02; Figure 5) across all studies in the meta-analysis. Heterogeneity was low and non-significant (I^2^=33%, *P*=.16). These findings represent an approximate increase of 300 kcal (95% CI 32 to 579) in energy expenditure per week. The quality of evidence was rated as being low based on being downgraded twice, once because of the high risk of bias identified and once because of the level of indirectness associated with the broad range of included interventions, comparators, populations, and settings.

#### Sedentary Behavior

**Figure 6:**
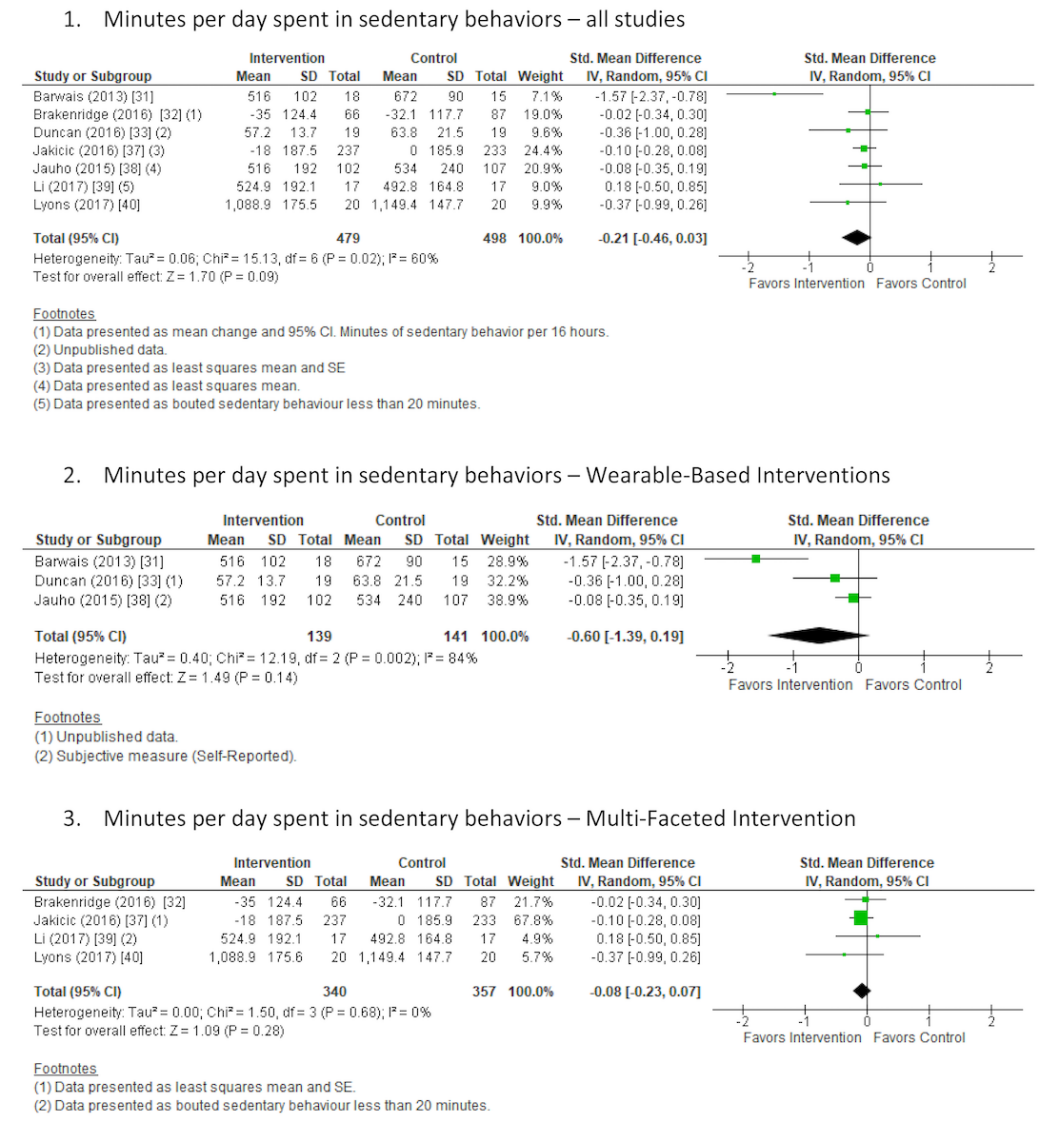
Forest plot of standardized mean difference of time spent in sedentary behaviors in studies comparing an intervention that included a consumer-based wearable activity tracker with a control group that did not utilize a consumer-based wearable activity tracker. Subgroup analysis was completed on studies that included wearable-based interventions compared with control and multifaceted interventions compared with control. Green square indicates the standardized mean difference for each individual study. Black square indicates the overall standardized mean difference for all studies.

Overall, 8 studies [29,31-33,37-40] reported changes in sedentary behavior. Furthermore, 2 studies [29,37] reported sedentary behavior as percentage of the day spent sitting, and 7 studies [31-33,37-40] reported minutes or hours of sedentary behavior per day. One study [39] reported sedentary behavior as bouts of greater than 20 min, and 1 study [32] reported minutes of sedentary behavior per 16 hours. Sedentary behavior was objectively measured except for 1 study [38], which utilized a self-reported questionnaire to obtain daily sitting time. A random-effects meta-analysis was completed on 7 studies that reported changes in sedentary behavior using SMD. One study [29] that reported sedentary behavior as percentage only was not included in the meta-analysis. For the 1 study [37] that reported sedentary behavior as percentage and hours per day, only hours per day were included in the analysis. There was a nonsignificant decrease in sedentary behavior following the intervention versus control comparator (SMD −0.21; 95% CI -0.46 to 0.03; *P*=.09; Figure 6) across all studies in the meta-analysis with a moderate and significant level of heterogeneity (I^2^=60%, *P*=.02). This finding represents approximately 37 min (95% CI −81 to 5 min) less spent in sedentary behavior. The quality of evidence was rated as very low based on being downgraded 3 times, once because of the high risk of bias identified, once because of the level of inconsistency associated with the observed heterogeneity, and once because of the level of indirectness associated with the broad range of included interventions, comparators, populations, and settings.

Further subgroup analysis was completed on studies that included a wearable-based intervention [31,33,38] compared with control and multifaceted interventions [32,37,39,40] compared with control. There was a nonsignificant decrease in sedentary behavior following both wearable-based and multifaceted interventions versus control comparator (SMD −0.60; 95% CI −1.40 to 0.19; *P*=.14 and SMD −0.08; 95% CI −0.23 to 0.07; *P*=.28, respectively; Figure 6) across the meta-analyses. These findings represent a decrease of approximately 115 min (95% CI −269 to 36 min) per day spent in sedentary behaviors for wearable-based interventions and a decrease of 13 min (95% CI −39 to 12 min) spent in sedentary behaviors for multifaceted interventions. High and significant heterogeneity (I^2^=84%; *P*=.002) was observed for the wearable-based analysis and low and nonsignificant (I^2^=0%; *P*=.68) for the multifaceted analysis.

## Discussion

### Principal Findings

This systematic review and meta-analysis summarizes the results of interventions that utilize a consumer-based wearable activity tracker to assist in the improvement of physical activity participation [21,22,29-54]. The results show a significant improvement in all measures of physical activity participation when compared with control groups, even when interventions were separated into wearable-based and multifaceted. However, intervention groups that were multifaceted in nature appeared to have a greater effect on physical activity participation when compared with control groups than those that included just the use of a consumer-based wearable activity tracker compared with control groups. No real differences in sedentary behavior were observed for either wearable-based or multifaceted interventions compared with control groups.

### Physical Activity Participation

Participants who received an intervention including a consumer-based wearable activity tracker demonstrated a significant improvement in daily steps, MVPA, and energy expenditure when compared with control groups. The quality of evidence was low for daily steps and energy expenditure and very low for MVPA. MVPA was the only physical activity outcome measure with a significant level of heterogeneity. This is most likely because of the inclusion of a greater range of intervention types when compared with other outcome measures and the method by which MVPA was measured, including 2 studies [30,48] that subjectively measured MVPA and another study [39] that presented MVPA data in bouts. Despite the low level of certainty, it is encouraging to see a significant positive intervention effect across all included measures of physical activity participation. A recent review [89] into the use of an electronic activity monitor system as an intervention modality also concluded that activity monitors have the potential to increase physical activity levels as did a review into the inclusion of an activity monitor in addition to a behavioral physical activity intervention for overweight and obese adults [90]. A small-to-moderate effect on physical activity participation was also reported by a review that looked at the effect of wearables and smartphone apps as an intervention modality [91].

An overall positive effect for consumer-based wearable activity trackers as an intervention tool was observed, even when interventions were separated into wearable-based only and multifaceted interventions. Subgroup analysis determined a larger effect size for interventions that were multifaceted (vs control) than for wearable-based (vs control) interventions. This suggests that consumer-based wearable activity trackers can be effective on their own, but when combined with other behavior change techniques, such as telephone counseling or group-based education, the improvement in physical activity participation is greater. However, the magnitude of the additive effect of each individual component of multifaceted interventions cannot be determined by meta-analysis. Only 3 of the included studies [34,47,54] directly compared wearable-based and multifaceted interventions. Two of these studies [34,47] offered financial incentives, with both studies reporting a significant improvement in daily step count for those receiving financial incentives compared with either control groups or those not receiving incentives. Financial incentives have previously been shown to be effective at increasing exercise adherence [92]. The third study [54] reported that adding personalized coaching to the use of a consumer-based wearable activity tracker resulted in sustained increases in physical activity behaviors over the 4-week intervention period. For MVPA, the observed heterogeneity was low and nonsignificant for the wearable-based analysis but high and significant for the multifaceted analysis. This indicates that the different types of included interventions contribute to the observed heterogeneity.

Two of the included studies [34,44] reported a significant increase in daily steps, 4 studies [30,34,36,53] reported a significant increase in MVPA, and 2 studies [45,47] reported a significant increase in energy expenditure for the intervention group compared with control. Most of the remaining studies reported a nonsignificant increase in physical activity participation. One study reported a reduction in daily steps [42], 1 study reported a reduction in MVPA participation [37], and 3 studies reported a reduction in energy expenditure [46,47,52] in at least one of the included intervention groups. Potential reasons why other included studies did not find a significant intervention effect or observed a reduction in physical activity participation may be because of several of the studies being pilot studies [29,30,40,47,52], which often have insufficient power because of small sample sizes [93]; a moderate-to-high loss to follow-up in some studies [29,32,33,37,42,43,46,48]; the type of intervention provided to the control group; the actual wear time of the activity tracker; the length of the interventions; and the use of subjective outcome measures. In addition, 8 studies [29,32,33,37,42,43,46,48] that reported physical activity outcome measures had greater than 20% loss to follow-up, with 3 of these studies reporting a reduction in physical activity participation. One study [32] reported a 56% loss to follow-up in the intervention group and 65% in the control group, and another study [43] had a large disparity in loss to follow-up between groups, with 47% loss in the control group compared with 11.7% loss in the intervention group. Intention-to-treat analysis was used in the statistical analysis of these studies, which is a cautious approach and minimizes the influence of loss to follow-up. Despite this, this style of analysis is less likely to show a positive treatment effect [94]. A variety of comparator interventions were utilized within the included studies, ranging from no contact in the form of usual care or waitlist comparators to participants being provided with behavioral group-based interventions or telephone counseling. Previously, both group behavioral interventions and telephone counseling have been demonstrated to be effective in increasing physical activity participation [14,95,96]. Actual wear time of the activity tracker varied, ranging from over 90% wear time [21,22] to all participants ceasing to wear the device by the end of the intervention [32] as the study design allowed self-directed wear of the activity tracker. The 2 studies that were 12 months or longer [32,37] reported lower adherence rates compared with shorter duration studies. Issues with long-term adherence to lifestyle and behavioral change interventions are well recognized [16,17,97]. Subjective, self-reported questionnaires were used to obtain participants’ daily energy expenditure in all included studies and MVPA in 2 included studies. Although the use of these types of questionnaires is widely accepted, self-reported questionnaires have been shown to be less robust in measuring energy expenditure and MVPA when compared with objective measures [98,99].

### Sedentary Behavior

Less time was spent in sedentary behavior for those receiving a consumer-based wearable activity tracker intervention compared with control groups; however, the finding was not significant, and the quality of the evidence was graded as very low [29,31-33,37-40]. Similar to the studies included in the physical activity participation analyses, a high risk of bias and serious level of indirectness was identified in the included studies. In addition, because of the observed level of heterogeneity, the quality of evidence was further downgraded. The included studies reported sedentary behavior in a range of formats, including percentage, minutes per day and per 16 hours, bouts of sitting time, and objective and subjective measurements, which could contribute to the observed level of heterogeneity. Only 1 included study [32] specifically aimed to reduce sitting time. One other study [40] utilized the idle alert feature of the consumer-based wearable activity tracker. Although a reduction of sitting time is often observed in interventions targeting physical activity promotion, interventions that specifically target sedentary behaviors are more effective [100-102]. The lack of specific focus on reducing sedentary behavior in 6 of the 8 included studies may have contributed to the nonsignificant finding. In contrast to the reported findings relating to physical activity participation, the subgroup analysis showed that the wearable-based interventions had a greater effect on reducing sedentary behaviors compared with control groups than the multifaceted interventions. This finding may be because none of the interventions included in the wearable-based meta-analysis exceeded 12 weeks in duration, whereas the multifaceted analysis included 2 studies that were 12 months or more in duration. Interestingly, the wearable-based interventions had a high and significant level of heterogeneity, whereas the heterogeneity was nonsignificant and low for the multifaceted interventions. This result was potentially because of the inclusion of 1 study [31] in the wearable-based meta-analysis that reported a 21% reduction in sitting time.

### Strengths and Limitations of Review

The current analysis incorporates a wide range of participant populations, ranging from younger to older adults as well as individuals that are apparently healthy to individuals with diagnosed chronic conditions. Previous reviews have focused on specific chronic condition populations [103] and patient subgroups such as overweight and obese adults [90]. A thorough systematic methodology was followed, and the inclusion of a meta-analysis allows for interpretation of the combined effects of including a consumer-based wearable activity tracker as part of a physical activity intervention. A wide range of physical activity interventions that differ in the way in which a consumer-based wearable activity tracker was utilized were included in the review. The benefits of using a consumer-based wearable activity tracker in addition to behavioral interventions have previously been demonstrated [90], meaning this review adds further support for the use of consumer-based wearable activity trackers in a range of different settings. In addition, the use of a consumer-based wearable activity tracker as a stand-alone intervention was examined, with the results indicating that even without supporting behavior change techniques, the use of a consumer-based wearable activity tracker could be effective in increasing physical activity participation. This may have clinical relevance as increased physical activity participation may lead to improvements in overall health.

Although the inclusion of a wide range of interventions and study populations had advantages in terms of general applications, the heterogeneity of the included study designs makes the comparison and synthesis of results difficult and lowers the overall quality of the evidence. The interventions used as comparators also differ greatly between included studies, once again making the comparison of results difficult.

### Conclusions and Practical Implications

Utilizing a consumer-based wearable activity tracker either as the primary component of an intervention or as part of a broader physical activity intervention has the potential to increase physical activity participation. Although the quality of evidence is low to very low, the included studies encompass a large age range and include males and females and a range of healthy and chronic condition populations. Although findings were not significant in all studies, short-term interventions utilizing a consumer-based wearable activity tracker generally resulted in increased physical activity participation. This suggests that consumer-based wearable activity trackers may be complementary to traditional intervention modalities such as group-based education and telephone counseling. The effects of physical activity interventions are generally short term, with ongoing contact from health professionals increasing long-term adherence to physical activity participation. Therefore, consumer-based wearable activity trackers have the potential to be included as an effective tool to assist health professionals to provide ongoing monitoring and support to patients with minimal resource expenditure. Further research to determine the effect of consumer-based wearable activity tracker independent of other traditional physical activity interventions would be beneficial as would investigations of the cost-effectiveness of consumer-based wearable activity tracker interventions. Given the potential novelty factor associated with the use of consumer-based wearable activity trackers, further investigation into their long-term usage and effectiveness would be useful to guide potential clinical applications and future recommendations.

## Appendix

Multimedia Appendix 1 Full search strategy.

Multimedia Appendix 2 Exclusion criteria.

Multimedia Appendix 3 Detailed summary of study design and baseline characteristics for all included studies.

Multimedia Appendix 4 GradePRO summary of findings table.

## Abbreviations

GRADE: Grades of Recommendation, Assessment, Development, and Evaluation
IQR: interquartile range
MeSH: Medical Subject Headings
MVPA: moderate and vigorous physical activity
PAM: Personal Activity Monitor
SMD: standardized mean difference
