# Supplementary material for: Consumer-Based Wearable Activity Trackers Increase Physical Activity Participation: Systematic Review and Meta-Analysis
Source: JMIR Mhealth Uhealth. 2019 Apr 12;7(4):e11819. doi: 10.2196/11819 (PMC6484266; doi:10.2196/11819)
Supplement: Multimedia Appendix 1 [file mhealth_v7i4e11819_app1.pdf]

CENTRAL (Including HTA)

#1 Activity Tracker

#2 Activity Trackers

#3 Fitbit

#4 wearable device

#5 wearable devices

#6 wearable technology

#7 fitness tracker

#8 fitness trackers

#9 Lifestyle education

#10 Behavio*r change

#11 Sedentary behavio*r

#12 step count

#13 Physical fitness

#14 Physical activity

#15 MeSH descriptor: [Sedentary Lifestyle] this term only

#16 MeSH descriptor: [Physical Fitness] this term only

#17 #1 or #2 or #3 or #4 or #5 or #6 or #7 or #8

#18 #9 or #10 or #11 or #12 or #13 or #14 or #15 or #16

#19 #17 and #18

Medline via Ovid

1. Activity traker.ab,ti.
2. Activity trackers.ab,ti.
3. Fitbit.ab,ti.
4. Wearable device.ab,ti.
5. Wearable devices.ab,ti.
6. Wearable technology.ab,ti.
7. Fitness tracker.ab,ti.
8. Fitness trackers.ab,ti.
9. *Physical Fitness/
10. Physical fitness.ab,ti.
11. Lifestyle education.ab,ti.
12. Behavio?r change.ab,ti.
13. Sedentary behavio?r.ab,ti.
14. Step count.ab,ti.
15. Physical activity.ab,ti.
16. Sedentary lifestyle.ab,ti.
17. *Sedentary Lifestyle/
18. 1 or 2 or 3 or 4 or 5 or 6 or 7 or 8
19. 9 or 10 or 11 or 12 or 13 or 14 or 15 or 16 or 17
20. 18 and 19

Embase via OVID

1. Activity traker.ab,ti.
2. Activity trackers.ab,ti.
3. Fitbit.ab,ti.
4. Wearable device.ab,ti.
5. Wearable devices.ab,ti.
6. Wearable technology.ab,ti.
7. Fitness tracker.ab,ti.
8. Fitness trackers.ab,ti.
9. *Physical Fitness/
10. Physical fitness.ab,ti.
11. Lifestyle education.ab,ti.
12. Behavio?r change.ab,ti.
13. Sedentary behavio?r.ab,ti.
14. Step count.ab,ti.
15. *Physical Activity/
16. physical activity.ab.ti
17. sedentary lifestyle.ab.ti
18. 1 or 2 or 3 or 4 or 5 or 6 or 7 or 8
19. 9 or 10 or 11 or 12 or 13 or 14 or 15 or 16 or 17
20. 18 and 19

PsychInfo via OVID

1. Activity traker.ab,ti.
2. Activity trackers.ab,ti.
3. Fitbit.ab,ti.
4. Wearable device.ab,ti.
5. Wearable devices.ab,ti.
6. Wearable technology.ab,ti.
7. Fitness tracker.ab,ti.
8. Fitness trackers.ab,ti.
9. *Physical Fitness/
10. Physical fitness.ab,ti.
11. Lifestyle education.ab,ti.
12. Behavio?r change.ab,ti.
13. Sedentary behavio?r.ab,ti.
14. Step count.ab,ti.
15. *Physical Activity/
16. physical activity.ab.ti
17. sedentary lifestyle.ab.ti
18. 1 or 2 or 3 or 4 or 5 or 6 or 7 or 8
19. 9 or 10 or 11 or 12 or 13 or 14 or 15 or 16 or 17
20. 18 and 19
