# Supplementary material for: Consumer-Based Wearable Activity Trackers Increase Physical Activity Participation: Systematic Review and Meta-Analysis
Source: JMIR Mhealth Uhealth. 2019 Apr 12;7(4):e11819. doi: 10.2196/11819 (PMC6484266; doi:10.2196/11819)
Supplement: Multimedia Appendix 2 [file mhealth_v7i4e11819_app2.pdf]

Exclusion Criteria

1. Not a human study population
2. Paediatric population
3. Wrong Intervention (didn’t include use of a wearable activity monitor)
4. Wrong Comparator
5. Wrong study deisgn
6. No control group
7. Ongoing study / no results reported
8. Conference abstract
9. Review paper
