# Supplementary material for: Consumer-Based Wearable Activity Trackers Increase Physical Activity Participation: Systematic Review and Meta-Analysis
Source: JMIR Mhealth Uhealth. 2019 Apr 12;7(4):e11819. doi: 10.2196/11819 (PMC6484266; doi:10.2196/11819)
Supplement: Multimedia Appendix 3 [file mhealth_v7i4e11819_app3.pdf]

Study	Duration	Participants	Intervention	Wearable based/ Multi-faceted	n	Age (yr)	BMI	Gender
Ashe, Winters et al.<br><br># [29]	6 months	Sedentary women (55-70yrs)	**Intervention** – Group based education + Fitbit One AT<br>**Control** – Group based education	Multi-faceted	20	63.9 (4.7)	29.9 (6.8)	F: 100%
Ashton, Morgan et al.<br><br># [30]	3 months	Sedentary young men (18-25yrs)	**Intervention** – Group based education + Jawbone AT<br>**Control** – Waitlist	Multi-faceted	50	21.1 (2.0)	25.5 (4.6)	M: 100%
Barwais, Cuddihy et al.<br><br># [31]	4 weeks	Sedentary Adults	**Intervention** – Gruve AT with online software<br>**Control** – Usual lifestyle	Wearable-based	33	27.7 (3.7)	WEIGHT: 78.0 (22.5)	F: 33.3%
Brakenridge, Fjdsoe et al.<br><br># [32]	12 months	Office workers	**Intervention** – Organisational wellness program + LUMOback AT<br>**Control** – Organisational wellness program	Multi-faceted	153	38.9 (8.0)	24.6 (3.4)	F: 46%
Cadmus-Bertram, Marcus et al.<br><br># [21]	16 weeks	Sedentary, post menopausal women with BMI >25	**Intervention** – Fitbit One AT + written information + phone call<br>**Control** – Pedometer + written information	Multi-faceted	52	59.9 (7.0)	29.1 (3.5)	F: 100%
Duncan, Vandelanotte et al.<br><br># [33]	9 weeks	Sedentary Adults (18-55yrs)	**Intervention** – Fitbit Charge HR AT+ study specific smartphone app<br>**Control** – Smartphone app	Wearable-based	64	35.8 (10.2)	27.3 (4.5)	F: 75%
Finkelstein, Haaland et al.<br><br># [34]	**6-month intervention**<br>6 month follow-up	Office workers (21-65yrs)	**Intervention 1** – Fitbit Zip AT + written information<br>**Intervention 2** – Fitbit Zip AT + written information + charity donation incentives<br>**Intervention 3** – Fitbit Zip AT + written information + cash incentives<br>**Control** – Written information	Wearable-based<br><br>Multi-faceted<br><br><br>Multi-faceted	800	35.5 (8.5)	WEIGHT: 66.1 (64.1-68.0) [a]	F: 53.7%
Hartman, Nelson et al. 2016<br><br># [35]	6 months	Women with BMI >27.5 & elevated breast cancer risk (40-75yrs)	**Intervention** – Fitbit One AT + regular phone calls<br>**Control** – Written information + 2 x brief phone calls	Multi-faceted	54	59.6 (5.7)	31.7 (3.5)	F: 100%

Hartman, Nelson et al. 2018<br><br># [36]	12 weeks	Sedentary, female breast cancer survivors<br>(21-85 yrs)	**Intervention** – Fitbit One AT + 2 x phone calls + regular emails<br>**Control** – Waitlist + regular emails (generic health topics)	Multi-faceted	87	57.2 (10.3)	27 (6.3)	F: 100%
Jakicic, Davis et al.<br><br># [37]	24 months	Adults with BMI >25 but <40<br>(25-40yrs)	**Intervention** – 0-6 months: group-based education. 7-24 months: FitCore AT + group-based education + weekly text messages + monthly phone calls<br>**Control** – As per intervention but no activity tracker provided	Multi-faceted	470	30.9 (18. – 35.9) [b]	1.2 (24.4 – 39.9) [b]	F: 71.1%
Jauho, Pyly et al.<br><br># [38]	3 months	Conscription aged men<br>(assessed in the year they turned 18)	**Intervention** – Polar Active AT<br>**Control** – Blinded Polar Active AT	Wearable-based	276	18.0 (0.9)	23.4 (4.5)	M: 100%
Li, Sayre et al.<br><br># [39]	1 month	Adults with knee OA<br>(50yrs +)	**Intervention** – Fitbit Flex AT + weekly phone calls + education sessions<br>**Control** – Waitlist	Multi-faceted	34	55.5 (8.6)	27.2 (4.7)	F: 82%
Lyons, Swarz et al.<br><br># [40]	12 weeks	Adults with BMI >25 but <35<br>(55-79yrs)	**Intervention** –Jawbone UP 24 AT + idle alert + weekly phone calls<br>**Control** – Waitlist	Multi-faceted	40	61.5 (5.6)	30.3 (3.5)	F: 85%
Martin, Feldman et al.<br><br># [41]	**P1: 2 wks**<br>P2: 2 wks	Adults<br>(18-69yrs)	**Intervention** – Fitbug AT + 3 x daily text messages<br>**Control** – Blinded Fitbug AT	Wearable-based	48	58 (8)	31 (6)	F: 46%
Melton, Buman et al.<br><br># [42]	**6 weeks**<br>8-week follow-up	College aged African American women<br>(18-24yrs)	**Intervention** –Jawbone UP AT + weekly emails<br>**Control** – Dietary intake self-monitoring through MyFitnessPal + weekly emails	Wearable-based	69	19.7 (1.8)	27.3 (6.8)	F: 100%
Pellegrini, Vebra et al.<br><br># [43]	6 months	Overweight adults<br>(21-55yrs)	**Intervention 1** – Group based education + BodyMedia Fit AT<br>**Intervention 2** – BodyMedia Fit AT + written information + phone calls<br>**Control** – Group education sessions	Multi-faceted<br><br>Multi-faceted	51	44.2 (8.7)	33.7 (3.6)	F: 86.3%
Poirier, Bennett et al	6 weeks	Office workers	**Intervention** – Pebble+ AT + online walking program + 4 x daily text messages<br>**Control** – Usual lifestyle	Wearable-based	265	39.9 (11.7)	-	F: 66%

# [44]								
Polzien, Jakicic et al.<br><br># [45]	12 weeks	Adults<br>(18-55yrs)	**Intervention 1** – BodyMedia Sensewear armband AT + individual counselling sessions<br>**Intervention 2 –** As per intervention 1 but AT only worn during weeks 1, 5 and 9<br>**Control** - Individual education sessions	Multi-faceted<br><br>Multi-faceted	57	41.3 (8.7)	33.1 (2.8)	F: 98%
Rogers, Lang et al.<br><br># [46]	6 months	Adults<br>(21-55yrs)	**Intervention 1** – BodyMedia Fit + written information + monthly phone calls<br>**Intervention 2** – BodyMedia Fit + smartphone app + written information + monthly phone calls<br>**Control** – Group based education	Multi-faceted<br><br>Multi-faceted	39	39.9 (11.5)	39.5 (2.8)	F: 79.5%
Shin, Yun et al.<br><br># [47]	12 weeks	Male students<br>(19-45yrs)	**Intervention** 1 – Fitmeter AT + study specific smartphone app + written information<br>**Intervention 2** – As per intervention 1 plus financial incentives for reaching step and body weight goals<br>**Control** – Written information	Wearable-based<br><br>Multi-faceted	35	27.8 (5.0)	29.8 (2.7)	M: 100%
Skrepnik, Spritzer et al.<br><br># [22]	90 days	Adults with knee OA eligible for Hylan G-F 20<br>(30-80yrs)	**Intervention** – Jawbone UP 24 AT + study specific smartphone app + written information<br>**Control** – Blinded Jawbone UP 24 AT + written information	Wearable-based	211	62.6 (9.4)	29.3 (3.7)	F:50.2%
Slootmaker, Chinapaw et al.<br><br># [48]	3 months	Office workers<br>(20-40yrs)	**Intervention** – Pam AT + interactive website<br>**Control** – Written information	Wearable-based	102	31.8(3.5)	25.2 (4.1)	F: 60%
Thompson, Kuhle et al. [c]<br><br># [49]	12 months (6-month cross over)	Sedentary, overweight older adults<br>(65-95yrs)	**Intervention** – Fitbit AT + written information + weekly phone calls + bi-monthly individual education sessions<br>**Control** – Blinded Fitbit AT	Multi-faceted	48	79.5 (7.0)	WEIGHT: 78.4 (13.5)	F: 81%
Thorndike, Mills et al.<br><br># [50]	**Phase 1: 6 weeks**<br>Phase 2: 6 weeks	Medical residents	**Intervention** –Fitbit AT + weekly email reminders with offer of incentives for wearing device<br>**Control** – As per intervention but with a blinded Fitbit AT	Multi-faceted	104	29 (23-37) [a]	24.1 (17.8-35.6) [a]	F: 54%

Unick, O'Leary et al.<br><br># [51]	6 months	Obese, sedentary adults<br>(21-55yrs)	**Intervention** – Group based education + Body Media Fit AT<br>**Control** – Group based education	Multi-faceted	29	42.4 (9.2)	45.0 (3.9)	F: 82%
Valle, Deal et al.<br><br># [52]	6 months	African American breast cancer survivors<br>(18yrs +)	**Intervention 1** – Individual education sessions, weekly emails, smartphone app paired with wi-fi scales. *No use of an activity tracker*<br>**Intervention 2** – As per intervention 1 + Withings AT<br>**Control** – waitlist	No AT used<br>Multi-faceted	35	53.1 (9.1)	34.0 (6.0)	F: 100%
van der Weegen, Verwey et al.<br><br># [53]	**6 months**<br>3-month follow-up	Primary care patients with T2DM or COPD<br>(40-70yrs)	**Intervention 1** – 4 x consultations with practice nurse. *No use of an AT*<br>**Intervention 2** – As per intervention 1 + unspecified AT + smartphone and web app<br>**Control** – Usual care	No AT used<br>Multi-faceted	199	57.9 (7.6)	29.4 (5.3)	F: 51.3%
Van Hoye, Boen et al. [c]<br><br># [54]	4-weeks	Adults aged<br>(19-67 yrs)	**Intervention 1** – Pedometer. *No use of an AT*<br>**Intervention 2 –** BodyMedia Sensewear armband AT<br>**Intervention 3 –** BodyMedia Sensewear armband AT + weekly sessions with a personal coach<br>**Control** – Baseline measures discussed in relation to national activity recommendations	No AT used<br>Wearable-only<br>Multi-faceted	221	42.4 (10.4)	27.1 (4.0)	F: 54.3%

AT = Activity tracker, [a] = range, [b] = 25th-75th percentile, [c] = studies not included in meta-analysis
