# Supplementary material for: Consumer-Based Wearable Activity Trackers Increase Physical Activity Participation: Systematic Review and Meta-Analysis
Source: JMIR Mhealth Uhealth. 2019 Apr 12;7(4):e11819. doi: 10.2196/11819 (PMC6484266; doi:10.2196/11819)
Supplement: Multimedia Appendix 4 [file mhealth_v7i4e11819_app4.pdf]

Summary of findings:

Wearable activity trackers compared to usual care in the promotion of physical activity

Patient or population: the promotion of physical activity
Setting:
Intervention: wearable activity trackers
Comparison: usual care

Outcomes	Anticipated absolute effects* (95% CI)		Relative effect (95% CI)	№ of participants (studies)	Certainty of the evidence (GRADE)	Comments
	Risk with usual care	Risk with wearable activity trackers				
Physical Activity (Steps/day) follow up: range 2 weeks to 12 months	-	-	-	2246 (12 RCTs)	⨁⨁◯◯ LOW [a,b]	A SMD of 0.24 represents an approximate increase of 627 steps (417 steps higher to 862 steps higher) per day.
Physical Activity (MVPA min/day) follow up: range 9 weeks to 24 months	-	-	-	2348 (11 RCTs)	⨁◯◯◯ VERY LOW [b,c,d]	A SMD of 0.27 represents an approximate increase of 40 minutes (22 minutes higher to 57 minutes higher) of MVPA per week
Physical Activity (kcal/wk) follow up: range 12 weeks to 6 months	-	-	-	369 (5 RCTs)	⨁⨁◯◯ LOW [b,e]	A SMD of 0.28 represents an approximate increase of 300 kcal (32 kcal higher to 579 kcal higher) of energy expenditure per week.
Sedentary Behaviour (min/day) follow up: range 1 months to 24 months	-	-	-	977 (7 RCTs)	⨁◯◯◯ VERY LOW [b,d,f]	A SMD of 0.20 represents an approximate decrease of 31 minutes (66 minutes lower to 4.5 minutes higher) in sitting time per day.

***The risk in the intervention group** (and its 95% confidence interval) is based on the assumed risk in the comparison group and the **relative effect** of the intervention (and its 95% CI).

CI: Confidence interval; **SMD:** Standardised mean difference

GRADE Working Group grades of evidence
High certainty: We are very confident that the true effect lies close to that of the estimate of the effect
Moderate certainty: We are moderately confident in the effect estimate: The true effect is likely to be close to the estimate of the effect, but there is a possibility that it is substantially different
Low certainty: Our confidence in the effect estimate is limited: The true effect may be substantially different from the estimate of the effect
Very low certainty: We have very little confidence in the effect estimate: The true effect is likely to be substantially different from the estimate of effect

Explanations

a. Unclear sequence generation in one study, no allocation concealment in one study and unclear allocation concealment in five studies, no blinding of participants in all studies, no blinding of outcome assessors in two studies and unclear blinding in five studies, greater than 20% loss to follow up in two studies, intention to treat analysis not used in one study and one study did not describe intention to treat analsysis and author conflicts of interest for one study.
b. Large variation in study populations, types of interventions utilised, outcome measures, length of intervention and

a broad age range across included studies.
c. Unclear sequence generation in one study, high risk of bias for allocation concealment in one study and unclear allocation concealment in three studies, no blinding of participants in all studies, unclear blinding of outcome assessors in five studies, greater than 20% loss in four studies, intention to treat analysis not used in one study, there was selective outcome reporting in two studies and an unclear reporting bias in one study due to the use of unpublished data.
d. Significant level of heterogeneity observed
e. Unclear sequence generation and allocation concealment in three studies, no blinding of participants in all studies, no blinding of outcome assessors in one study and unclear blinding in four studies, greater than 20% loss to follow up in two studies.
f. Unclear allocation concealment in three studies, no blinding of participants in all studies, no blinding of outcome assessors in two studies and unclear blinding in three studies, greater than 20% loss to follow up in three studies and one study did not use intention to treat analysis, unclear reporting bias due to the use of unpublished data.
